# Applications and efficacy of minoxidil in dermatology

**DOI:** 10.1002/ski2.472

**Published:** 2024-11-24

**Authors:** Ramadan S. Hussein, Salman Bin Dayel, Othman Abahussein, Abeer Ali El‐Sherbiny

**Affiliations:** ^1^ Department of Dermatology College of Medicine Prince Sattam Bin Abdulaziz University Al‐Kharj Saudi Arabia; ^2^ Department of Medical Laboratory College of Applied Medical Sciences Prince Sattam Bin Abdulaziz University Al‐Kharj Saudi Arabia

## Abstract

Minoxidil, originally developed as an antihypertensive medication, has evolved into a versatile therapeutic agent within dermatology, notably for its effectiveness in promoting hair growth. Despite its widespread use, understanding its precise mechanism of action remains a challenge. This paper addresses this gap by providing a comprehensive review of pharmacological properties, action mechanisms, clinical effectiveness and side effects associated with topical minoxidil. Furthermore, it highlights emerging trends and applications, including its role in treating androgenetic alopecia, its potential for alopecia areata management and its utilization in combination therapies. Additionally, this paper explores novel applications in scar treatment and wound healing. By synthesizing current knowledge and insights, this review adds clarity to the diverse applications of minoxidil, providing valuable guidance for clinicians and researchers aiming to optimize its therapeutic use in dermatology.

**What is already known about this topic?**

- Minoxidil was originally developed as an antihypertensive agent but is now widely used in dermatology for promoting hair growth. It has proven clinical efficacy in treating conditions such as androgenetic alopecia. However, despite its extensive use, the exact mechanism of action of topical minoxidil remains unclear.

**What does this study add?**

- This study provides a detailed review of the pharmacological mechanisms, clinical effectiveness, and side effects of topical minoxidil, while also exploring its emerging uses beyond hair growth, such as in scar treatment and wound healing. It highlights new applications and combination therapies, offering a comprehensive synthesis that can guide clinicians and researchers in optimizing its therapeutic potential across a broader range of dermatological conditions.

## 1 INTRODUCTION

Minoxidil stands as an enduring testament to the evolution of dermatological therapeutics, tracing its origins from an antihypertensive agent to a cornerstone in addressing diverse dermatological concerns. Initially recognized for its vasodilatory effects in managing hypertension, its serendipitous discovery of stimulating hair growth sparked a transformative journey within dermatology.^1^

Originally introduced in the 1970s as an oral antihypertensive medication, the subsequent revelation of its unintended yet remarkable side effect on hair growth inaugurated a paradigm shift. This unexpected finding paved the way for the development of topical formulations, heralding a new era in treating androgenetic alopecia (AGA).^2^

The formulation of minoxidil, primarily as a topical solution or foam, revolutionized its application in dermatology. Understanding its mechanisms of action, including its vasodilatory properties, modulation of potassium channels and effects on hair follicle cycling has been instrumental in elucidating its diverse therapeutic roles beyond its initial indication.^3^

The widespread adoption of minoxidil in dermatology emanates from its efficacy in arresting hair loss and stimulating regrowth in AGA. Beyond this cornerstone application, its utility has extended across various dermatological domains, showcasing efficacy in conditions ranging from alopecia areata (AA) to cutaneous vascular disorders and inflammatory skin conditions.^4^

The rationale underlying its pervasive use lies not only in its clinical effectiveness but also in its relatively favourable safety profile, making it a versatile choice for a broad spectrum of patients. Its mechanisms, though not entirely elucidated in every aspect, have paved the way for innovative treatment strategies and have spurred research into novel applications within dermatology.^5^

This review aims to delve into the comprehensive landscape of minoxidil's multifaceted utility within dermatology, encompassing its mechanisms, efficacy, safety and emerging trends, thereby providing a comprehensive understanding of its diverse applications across various dermatological conditions.

## 2 MECHANISMS OF ACTION AND PHARMACOLOGICAL INSIGHTS

Understanding the intricate mechanisms governing minoxidil's actions and its pharmacokinetic attributes is crucial for optimizing its therapeutic efficacy while mitigating potential adverse effects.

### 2.1 Potassium channel modulation and beyond

An in‐depth exploration of minoxidil's primary mechanism unveils its action as a potassium channel opener, a process believed to stimulate hair follicles and induce vasodilation. By opening potassium channels, minoxidil is thought to hyperpolarize cell membranes, leading to increased blood flow around hair follicles.^6^ This augmented blood supply is hypothesized to support hair growth by prolonging the anagen phase of hair cycle. Additionally, vasodilation potentially aids in delivering essential nutrients and oxygen to the follicles, contributing to their revitalization and promoting hair regrowth.^5^

Insights into additional molecular pathways influenced by minoxidil suggest its involvement beyond potassium channel opening. Studies indicate that minoxidil may impact various pathways linked to hair growth including prostaglandin synthesis, endothelin‐1 modulation and vascular endothelial growth factor expression.^7^, ^8^ These multifaceted interactions suggest that minoxidil's therapeutic actions might extend beyond its known vasodilatory effects, potentially involving complex signalling cascades influencing hair follicle growth and regeneration.^8^

### 2.2 Absorption dynamics and bioavailability

Upon topical application, minoxidil is absorbed through the skin, reaching systemic circulation. It demonstrates limited systemic absorption and most of the applied dose remains localized at the site of application. Roughly 1.4% of minoxidil applied topically on a healthy scalp is absorbed, with increased absorption linked to the concentration of the drug, how often it is applied and any damage to the protective function of outer layer of skin.^9^ Less than 1% of minoxidil applied on the scalp is absorbed systemically. Minoxidil does not attach to proteins in the blood or penetrate the blood–brain barrier. Once absorbed, minoxidil undergoes metabolism primarily in the liver where it is converted into its active form, minoxidil sulphate. This metabolite is responsible for the vasodilatory effects associated with minoxidil. Around 95% of minoxidil and its byproducts absorbed into the bloodstream are eliminated through the kidneys within 4 days.^10^

Understanding the absorption profiles of minoxidil holds significant implications for its therapeutic efficacy and dosing strategies. The limited systemic absorption of topically applied minoxidil influences its localized action, emphasizing its suitability for dermatological conditions. Tailoring dosing regimens to optimize absorption and sustain therapeutic concentrations at the target site is pivotal. Formulation considerations such as delivery systems that enhance skin penetration or sustained release can potentially prolong its local availability, improving treatment efficacy.^11^

## 3 MINOXIDIL IN HAIR DISORDERS

Minoxidil, a pivotal agent in the management of various hair disorders, exhibits distinct efficacy profiles and considerations across diverse conditions, ushering in an era of multifaceted treatment approaches. The FDA has granted approval for the application of topical minoxidil in treating AGA. Furthermore, it has been employed off‐label to address various hair conditions such as scarring alopecia, AA and disorders related to the hair shaft. Additionally, its off‐label usage extends to enhancing hair growth in different body regions including the eyebrows and beard.^6^

### 3.1 Treatment of alopecia

Summary of minoxidil applications in alopecia (Table 1).

**TABLE 1:** Summary of minoxidil applications in alopecia.

Condition	Form	Concentration	Efficacy	Side effects	Notes
Androgenetic alopecia^12^, ^13^	Topical	2%/5%	Superior to placebo, higher efficacy at 5%	Contact dermatitis, irritation	5% foam better tolerated
Androgenetic alopecia^14^	Oral	Variable	Effective for non‐responders to topical form	Systemic side effects	Often combined with spironolactone
Alopecia areata^15^, ^16^	Topical	3%/5%	Moderate efficacy, better at higher doses	Mild local effects	Limited efficacy in widespread cases
Alopecia areata^17^	Oral	5 mg	Higher rates of regrowth, but significant side effects	Systemic side effects	Used in resistant cases
Scarring alopecia^18^	Topical	2%/5%	Beneficial in off‐label use	Contact dermatitis, irritation	Requires combination with other treatments
Eyebrows and beard^19^	Topical	2%/5%	Effective in enhancing growth	Mild local effects	Off‐label use

### 3.2 Other hair disorders

Exploring minoxidil's application in various hair disorders beyond its conventional use involves gaining insights into its potential efficacy in conditions like telogen effluvium, traction alopecia and frontal fibrosing alopecia (FFA).^20^

A recent retrospective analysis of 36 chronic telogen effluvium (CTE) female patients who received oral minoxidil daily doses ranging from 0.25 to 2.5 mg demonstrated a significant decrease in the shedding of hair at both 6 and 12 months. Certain patients experienced adverse effects, including dizziness, slight facial hypertrichosis and changes in blood pressure.^21^

The distinctive quality of being eversible and non‐scarring during the initial phases of traction alopecia could benefit from the use of minoxidil. In two cases where topical triamcinolone yielded no response over 1 year, substantial hair growth was observed with the use of 2% minoxidil alone.^22^

FFA, a form of scarring alopecia primarily affecting the frontal hairline, presents challenges in treatment. In a case review, half of FFA patients (*n* = 7) exhibited slow disease progression when subjected to combination therapy involving applying 2% minoxidil twice daily along with systemic steroids or finasteride.^18^

### 3.3 Factors influencing efficacy

Considering patient selection, response variability and prognostic factors across various hair disorders is critical when evaluating the efficacy of minoxidil. Patient selection involves identifying individuals who are most likely to benefit from minoxidil treatment based on factors such as the type and severity of hair disorder, the extent of hair loss, underlying health conditions and individual responsiveness to treatment. Understanding which patients are more likely to respond positively to minoxidil aids in optimizing treatment outcomes.^23^, ^24^

Response variability refers to the diversity in individual responses to minoxidil treatment. Factors such as genetic predisposition, hormonal influences, scalp conditions and adherence to treatment can lead to variations in how individuals respond to minoxidil. Analyzing response variability helps in predicting and managing differing outcomes among patients.^23^, ^25^

Prognostic factors across different hair disorders encompass a range of considerations influencing the prognosis or expected course of hair disorder. These factors may include the duration and stage of condition, the presence of underlying medical conditions, lifestyle factors and the potential for interactions with other treatments or medications. Identifying and understanding these prognostic factors aids in predicting treatment outcomes and guiding therapeutic decisions.^26^

### 3.4 Combination therapies and comparative effectiveness studies

Studying combination therapies helps elucidate whether the integration of minoxidil with other treatments leads to additive or synergistic effects that surpass the outcomes achieved with monotherapy. Additionally, it explores the potential for reducing dosages or adverse effects associated with individual treatments while maintaining or enhancing efficacy. Researchers and clinicians investigate the potential synergies between minoxidil and complementary therapies such as finasteride, platelet‐rich plasma (PRP), microneedling (a technique involving tiny needles to create micro‐injuries in the scalp), low‐level laser therapy (LLLT) or certain topical formulations. This assessment aims to understand how combining minoxidil with other modalities may produce augmented effects on hair regrowth, hair density or the reversal of hair loss.^27^, ^28^

Efforts have been undertaken to utilize oral minoxidil for patients with FPHL or AGA who found conventional treatments unsatisfactory. Combining a low‐dose of 2.5 mg minoxidil with spironolactone at 25 mg in FPHL patients demonstrated favourable outcomes, leading to hair shedding reduction and hair density improvement. The mean severity score dropped to 2.3 after 6 months and 2.6 after 12 months. Some mild adverse effects were noted such as postural hypotension, urticaria and facial hypertrichosis. Crucially, the study did not observe any significant alteration in blood pressure.^14^

Olsen and colleagues showed that combining previous systemic corticosteroid usage (lasting more than 6 weeks) with the application of 2% minoxidil (three times daily) resulted in a better outcome, fostering sustained hair growth compared to using 2% minoxidil alone in extensive cases of AA.^29^

Efficacy of combination treatment of minoxidil and Plasma PRP, LLLT and microneedling was shown in Table 2.

**TABLE 2:** Combination treatment of minoxidil with PRP, LLLT and microneedling.

Authors	Experimental design	Number of patients	Experimental groups	Results	Adverse effects
Alves and Grimalt^30^	Split scalp study comparing 5% topical minoxidil versus PRP injection	13	Minoxidil monotherapy versus minoxidil + PRP	Combination therapy led to a notable rise in hair density and average hair count in comparison to using minoxidil alone. Small sample size and limited follow‐up (6 months)	Not specified
Pakhomova and Smirnova^31^	Three‐arm study comparing topical minoxidil 5% with PRP	69	Topical minoxidil monotherapy versus PRP monotherapy versus combination therapy	Combination therapy significantly outperformed PRP monotherapy, nearly tripling overall hair density, with similar synergistic benefits observed in other studies	Not specified
Gentile et al.^32^	Comparison of LLLT with PRP and microneedling	23	LLLT + PRP + microneedling	Combined treatment increased mean hair density at 58 weeks for all patients compared to baseline, proving effective and safe despite the absence of a control group	Not specified
Shah et al.^33^	Comparison of PRP with microneedling and minoxidil	50	Topical minoxidil monotherapy versus PRP + microneedling	Combination therapy resulted in the highest satisfaction and improvement ratings from both patients and clinicians	Not specified
Yepuri and Venkataram^34^	Trial of PRP and microneedling combination therapy	60	PRP + microneedling	Over 80% of participants experienced at least 40% improvement in hair growth after four sessions	Not specified
Jha et al.^35^	Retrospective study evaluating minoxidil, PRP, and microneedling	93	Topical minoxidil monotherapy versus PRP + topical minoxidil versus PRP + topical minoxidil + microneedling	Triple combination therapy showed the greatest terminal to vellus hair ratio, the highest number of negative hair pull tests, and the most significant improvement in hair growth	Not specified

Abbreviations: LLLT, low‐level laser therapy; PRP, platelet‐rich plasma.

In general, the accumulating evidence suggests that combining multiple therapies yields superior results compared to individual treatment approaches. Considering the favourable safety profiles of topical minoxidil, LLLT, microneedling and PRP, patients undergoing combined therapy are not exposed to an elevated risk of adverse effects.^34^, ^35^

## 4 DERMATOLOGICAL APPLICATIONS BEYOND HAIR DISORDERS

While predominantly acclaimed for its efficacy in hair disorders, minoxidil's therapeutic reach extends beyond, delving into a diverse array of dermatological conditions and showcasing its versatility and potential in addressing broader skin concerns.

### 4.1 Minoxidil in the management of cutaneous scars and wound healing

Minoxidil, a commonly prescribed medication for hair loss, has garnered attention for its potential therapeutic role in scar treatment. Its mechanism of action involves enhancing blood flow to the affected area, promoting tissue repair and regeneration. A 1991 study by Timo et al. revealed that minoxidil affects human skin fibroblasts, specifically reducing lysyl hydroxylase activity at the transcriptional level. This inhibition may impede lysyl hydroxylase synthesis which is crucial for collagen production.^36^ Minoxidil also inhibits cell proliferation without inducing toxicity leading to a decrease in DNA synthesis. Given collagen's significance in fibroblasts and lysyl hydroxylase's role in collagen biosynthesis, these combined effects suggest minoxidil's potential as an antifibrotic agent, particularly for skin conditions linked to collagen accumulation.^37^

In a 2021 study, minoxidil exhibited the ability to impede cell proliferation in clubfoot fibroblast‐like cells, resulting in notable alterations in extracellular matrix collagen content. Minoxidil also reduced collagen type I fibre deposition, structural maturation and assembly along with a concentration‐dependent decrease in cell‐mediated contraction of collagen gel lattices.^38^ These findings suggest that minoxidil could serve as a supplementary pharmacological treatment to alleviate fibrosis and diminish collagen in scar lesions when locally administered.

Despite ongoing research on antifibrotic medications for various conditions, limited attention has been given to exploring minoxidil's potential in scar treatment. Further investigation is crucial to fully understand this potential and its application in addressing acne scars.^39^

### 4.2 Role of minoxidil in wound healing

Minoxidil, originally known for its vasodilatory properties and hair growth stimulation, was expected to inhibit fibroblast activity and collagen synthesis based on in vitro studies.^40^ However, this in vivo study did not replicate these findings, as founded by Khazaeli et al. study, which introduced a novel topical gel formulation of minoxidil to explore its effects on an experimental induced a second‐degree skin burn on the back of wistar rats’ model wound healing. Despite prior evidence of minoxidil's potential as an inhibitor of wound contraction and scarring, the results showed no significant changes in collagen content or wound contraction between minoxidil‐treated animals and control animals in a burn wound model. Additionally, while minoxidil did induce angiogenesis in the burned area of the skin its mechanism of action remains uncertain. Limitations of the study include the lack of pharmacokinetic and pharmacodynamic data for the gel formulations and the inability to explore combination therapies due to logistical constraints.^41^

## 5 ADVERSE EFFECTS AND THEIR MANAGEMENT STRATEGIES

The identification and understanding of both common and rare adverse effects associated with minoxidil usage are vital for ensuring patient safety and informed decision‐making. Common side effects, such as scalp irritation or increased hair shedding during the initial phase of treatment, are typically transient and manageable. However, recognizing rare adverse effects like allergic reactions, severe scalp irritation or unwanted hair growth in unintended areas is equally important.^42^ The occurrence of excessive hair growth, known as hypertrichosis, is influenced by the concentration of minoxidil used, with individuals treated with 5% minoxidil solution experiencing the highest frequency of unwanted hair growth. This phenomenon is more prevalent among female patients compared to male patients, and while the exact reason remains unclear, some female patients may possess a greater number of hair follicles sensitive to minoxidil. After discontinuation of minoxidil treatment, spontaneous resolution of hypertrichosis typically begins on the face and arms within 1–3 months, followed by the legs within 4–5 months.^43^ There had been an assumption that excessive application of minoxidil topically could lead to its absorption into the bloodstream, causing excessive hair growth in untreated areas. However, there is no evidence to support this claim, as application of topical minoxidil twice daily has been found to have no systemic side effects such as low blood pressure, abnormal heart rate or weight gain.^44^ Overall, topical minoxidil is considered safe and effective for various hair disorders providing positive outcomes. Thorough delineation and awareness of these potential adverse effects empower healthcare providers to counsel patients effectively and monitor them for any unexpected reactions, ensuring safer and more informed use of minoxidil.^45^

Mitigating adverse reactions to minoxidil involves a multifaceted approach that begins with comprehensive patient counselling. Educating individuals about potential side effects, emphasizing proper application techniques and highlighting the expected timeline for results helps manage expectations and minimizes anxiety. Encouraging patch tests and advising gradual application during the initial stages can reduce the likelihood of irritation. Proactive management includes regular follow‐ups to monitor progress and address any emerging side effects promptly. Healthcare providers should encourage open communication ensuring patients feel comfortable reporting any concerns. Adjustment of dosage or formulation and recommending supportive treatments to alleviate side effects such as using moisturizers to counter scalp dryness can enhance treatment adherence and comfort. Ultimately, fostering a supportive environment through education, vigilant monitoring and responsive care significantly contributes to ensuring patient safety and treatment compliance in minoxidil therapy.^44^, ^45^

## 6 FUTURE DIRECTIONS AND EMERGING TRENDS

Anticipating potential advancements, exploring uncharted therapeutic avenues and forecasting the evolution of minoxidil's role within dermatology highlight the dynamic landscape shaping its future applications as shown in Table 3.

**TABLE 3:** Advancements in minoxidil formulations and ongoing research.^9^, ^46^, ^47^

Advancements in minoxidil formulations and delivery systems	Unexplored therapeutic avenues and ongoing research
Innovations and technological progress	Investigating novel applications
Continual advancements in minoxidil formulations, such as gels, nanoparticles and sustained release systems are revolutionizing treatment approaches, augmenting efficacy and improving patient convenience	Minoxidil holds potential in wound healing, scar management, alopecia areata, dermatological disorders, cosmetic applications and neurology
Gel formulations	Clinical exploration
Innovations aim to enhance drug delivery and absorption, offering improved skin penetration and better localized effects. Gels provide a non‐greasy easily spreadable application experience	Rigorous studies are essential to establish efficacy, safety and optimal usage in these uncharted therapeutic domains
Nanoparticle technology	
Utilizing nanoparticles facilitates controlled release, prolonging drug effects and optimizing delivery to hair follicles and targeted areas	
Sustained release systems	
Developing mechanisms extend drug action, reducing the need for frequent applications and maintaining therapeutic levels over time	
Enhanced patient experience	
Formulations with improved textures and reduced odour contribute to a more pleasant treatment experience	
Tailored delivery systems	
Customized systems target specific areas effectively while minimizing systemic absorption and side effects	
Future prospects	
Ongoing research explores innovative ways to optimize minoxidil delivery, promising greater efficacy and improved patient outcomes	

## 7 CONCLUSION

Across various dermatological conditions, minoxidil demonstrates promising potential but exhibits varying degrees of efficacy. In AGA, it stands as a cornerstone treatment showcasing moderate effectiveness in hair regrowth and maintenance. However, its efficacy in conditions like AA or inflammatory skin conditions remains less established, requiring further exploration through rigorous studies.

Safety profiles generally align, emphasizing its overall well‐tolerated nature particularly when used topically. Adverse reactions are typically mild, localized and transient minimizing concerns of systemic effects. However, larger‐scale standardized research across diverse dermatoses is essential to solidify these conclusions and ensure comprehensive insights into minoxidil's therapeutic spectrum.

## CONFLICT OF INTEREST STATEMENT

The authors declare no conflicts of interest.

## AUTHOR CONTRIBUTIONS

**Ramadan S. Hussein**: Data curation (equal); writing—original draft (equal); writing—review & editing (equal). **Salman Bin Dayel**: Conceptualization (equal); formal analysis (equal). **Othman Abahussein**: Supervision (equal); validation (equal); visualization (equal). **Abeer Ali El‐Sherbiny**: Conceptualization (equal); software (equal); writing—review & editing (equal).

## ETHICS STATEMENT

Not applicable.

## PATIENT CONSENT

Not applicable.

## Data Availability

The data underlying this article will be shared on reasonable request to the corresponding author.
